# Self-reported adherence and pharmacy refill adherence are both predictive for an undetectable viral load among HIV-infected migrants receiving cART

**DOI:** 10.1371/journal.pone.0186912

**Published:** 2017-11-09

**Authors:** Sabrina K. Been, Elif Yildiz, Pythia T. Nieuwkerk, Katalin Pogány, David A. M. C. van de Vijver, Annelies Verbon

**Affiliations:** 1 Department of Internal Medicine, division of Infectious Diseases, Erasmus University Medical Center, Rotterdam, The Netherlands; 2 Department of Medical Microbiology and Infectious Diseases, Erasmus University Medical Center, Rotterdam, The Netherlands; 3 Department of Medical Psychology, Academic Medical Center, Amsterdam, The Netherlands; 4 Department of Internal Medicine, Maasstad Hospital, Rotterdam, The Netherlands; 5 Viroscience department, Erasmus University Medical Center, Rotterdam, The Netherlands; Universita degli Studi di Roma Tor Vergata, ITALY

## Abstract

HIV-infected migrants were shown to have poorer treatment outcomes than Dutch HIV-infected patients, often due to worse treatment adherence. Self-reported adherence would be an easy way to monitor adherence, but its validity relative to pharmacy refill adherence has not been extensively evaluated in migrants. All HIV-infected migrants older than 18 years and in care at the two Rotterdam HIV-treatment centers were eligible. Refill data with leftover medication (PRL) (residual pill count) were obtained from their pharmacies up to 15 months prior to inclusion. Self-reported adherence to combination Antiretroviral Therapy was assessed by four questions about adherence at inclusion. Additionally, risk factors for pharmacy refill non-adherence were examined. In total, 299 HIV-infected migrants were included. Viral load (VL) was detectable in 11% of the patients. Specificity of PRL was 53% for patients with an adherence of 100% and decreased with lower cut-off values. Sensitivity and negative predictive value (NPV) were 68% and 15% and increased with lower cut-off values. Positive predictive value (PPV) was around 93% for all cut-off values. Using the self-reported questions, 139 patients (47%) reported to be adherent. Sensitivity was 49% and specificity was 72%. PPV and NPV were 95% and 13%. No risk factors for pharmacy refill non-adherence were found in multivariable analyses. Both PRL and self-reported adherence, can predict undetectable VL in HIV-infected migrants. PPV and NPV are similar for both methods. This study shows that using four self-reported items is sufficient to predict adherence which is crucial for optimal clinical outcome in HIV-infected migrants.

## Introduction

Treatment adherence is one of the strongest predictors of virological failure, development of drug resistance, disease progression and death [1]. Poor adherence to combination antiretroviral therapy (cART) is common in both developing and developed nations. It was found in around 20% of HIV-infected patients in Africa and in around 14% in the United Stated of America [2–4]. It is not clear what the influence of migration from developing countries to developed countries is on adherence and outcome. In Europe, migrant people living with HIV (MPLWH) have worse treatment outcomes than non-migrant patients living with HIV [5–9]. Although lack of adherence could not be demonstrated as a reason for virological failure in one study [6], factors associated with poor adherence such as younger age, high alcohol consumption, presence of psychiatric co-morbidity, stigma, lack of self-efficacy or social support are frequently found in migrants [6, 10, 11]. With the strongly increasing migration from Africa and the Middle East to Europe it is pivotal to determine adherence levels with the goal to intervene when adherence is poor in order to improve treatment outcome in MPLWH.

Treatment adherence of HIV-infected patients can be measured in several ways. Pharmacy refill data have been shown to be a useful tool in both developing and developed nations [2, 12–14]. Calculation of pharmacy refill adherence is most practical in closed pharmacy systems where information about dispensed medication can be retrieved from a single registry. In the absence of a closed pharmacy system, calculation of pharmacy refill adherence is more laborious because information retrieved from different pharmacies need to be combined. Although pharmacy refill data are valuable on a population level, it seems to be less suitable to measure adherence on an individual patient level. Self-reported adherence questions, administered either via questionnaires or via interviews, have previously been shown to be a practical adherence assessment method [6, 14]. However, it is well established that self-reports may overestimate adherence levels compared with more objective measures, which is attributed to recall bias and social desirability bias [15]. The validity of self-reported adherence questions administered among MPLWH in Europe might be influenced by cultural differences. This could facilitate social desirability bias.

Since retention in care and treatment adherence are crucial for a good outcome in HIV-infected patients [12, 16], and MPLWH were previously shown to have worse HIV treatment outcomes and adherence levels, valid and practical adherence measures are needed to identify persons in need of additional adherence support. We therefore assessed two methods, pharmacy refill adherence and self-reported adherence, in order to determine their comparative predictive value for undetectable viral load in a large group of MPLWH from different geographical origins. In addition, we examined risk factors for non-adherence based on pharmacy refill data calculation.

## Methods

### Study population

Patients included in the ROtterdam ADherence (ROAD) project, a study aiming to increase adherence in first and second generation adult immigrant people living with HIV (The Nederlands National Trial Register Number (NTR)—NTR4941) were recruited at outpatient clinics in the two Rotterdam HIV treatment centers (Erasmus University Medical Center and Maasstad Hospital) between November 2012 and July 2013 [11]. In these HIV treatment centers, 52% of the PLWH in care in 2014 were first generation immigrants (data not shown). Participants had to be sufficiently fluent in at least one of the following languages: Dutch, English, French, Spanish, or Portuguese.

This study focuses on a sub population of the 352 patients included in the ROAD project. Patients using cART for >6 months prior to inclusion signed informed consent allowing the investigators to consult their pharmacies about medication refills and to check the patients’ medical files for information about the prescribed start and stop of cART regimens, and viral load results. Additional relevant clinical and socio-demographic characteristics, and psychosocial variables were collected via an interviewer administered questionnaire [11]. An undetectable viral load (VL) was defined as HIV-1 RNA or HIV-2 RNA <50 copies/ml.

The study was approved by the Institutional Review Boards of the Erasmus University Medical Center and the Maasstad Hospital, Rotterdam.

### Study design and data collection

#### Collecting pharmacy refill data

In the Netherlands, there is no closed pharmacy system. Therefore, names of the pharmacies from which antiretroviral medication was collected were given by the participants when they attended the outpatient clinic for a routine visit. In the Netherlands, patients who are stable on cART usually collect their antiretroviral drugs at the pharmacy every 90 days. Pharmacy refill data was collected at one time point. We sent a fax to each patient’s pharmacy to ask for information regarding the type of antiretroviral medication, pills supplied, prescribed daily dose, date of supply up to 15 months prior to inclusion. A copy of the signed informed consent was provided along with the request. Reminders per email and fax were sent and if necessary, pharmacies were contacted by telephone.

#### Pharmacy refill adherence calculation

For our analyses, we assumed that medication was taken as prescribed until the supply was finished. Calculation of adherence with pharmacy refill data was done taking leftover medication (residual pill count) into account, a method that substantially limits misclassification of adherence [13]. In short, a period of 3 months prior to inclusion (90 days) was used as the episode to monitor adherence. Pharmacy refill adherence was calculated by determining the average supply percentage of all cART during this 90 days episode. The 12 months (360 days) prior to this episode were used to calculate the leftover medication. Antiretroviral regimen changes were taken into account. For the calculation of leftover medication, we assumed that patients had leftover medication if medication was refilled while the number of pills dispensed at a former refill divided by the prescribed daily doses and multiplied by the days since the previous refill was higher than zero, except when the patient changed his or her regimen. The residual number of pills was rolled over to the next refill period leading to the following formula for pharmacy refill adherence with leftover medication:

Pharmacyrefilladherencewhenleftovermedicationisincluded(PRL)(%)=(Leftovermedicationfrompreviousrefills+CollectedmedicationatrefillDaysbetweentworefills*Dailydose)*100

If the supply of pills for a refill period extended beyond the 90-day episode or the date of a switch, we truncated adherence at 100%. Additionally, we investigated the number of patients who would be misclassified as ‘non- adherent’ when leftover medication was not taken into account, using the following formula to determine pharmacy refill adherence:

Pharmacyrefilladherencewhenleftovermedicationisnotincluded(PR)(%)=(CollectedmedicationatrefillDaysbetweentworefills*Dailydose)*100

#### Self-reported adherence

Self-reported adherence was measured by four items via an interviewer administered questionnaire at the day of inclusion. The questionnaire was available in 4 languages: Dutch, English, French, and Spanish. The first two items measured recollection of adherence: Q1: ‘Thinking about the past four weeks, how would you rate your ability to take all your medications as your doctor prescribed them?’ (6-point Likert scale ranging from 1 = ‘very poor’ to 6 = ‘excellent’) and Q2: ‘Thinking about the past four weeks, how often did you take all your HIV antiretroviral medications as your doctor prescribed them?’ (5-point Likert scale ranging from 1 = ‘none of the time’ to ‘all of the time) [17]. Item three measured the adherence over the past week: Q3: ‘How many days in the past week did you take all anti-HIV medicines that were prescribed?’ (‘5-point Likert scale ranging from 1 = ‘not on day’ to 5 = ‘all 7 days’) [15, 18, 19]. The last item measured the most recent missed dose: Q4: ‘When was the last time you missed any of your anti- HIV medications?’ (6-point Likert scale ranging from 1 = ‘ within the past week’ to ‘never missed’ [20]. For the calculation of adherence, a patient was classified as ‘adherent’ if they had the following answers:

- Q1: ‘very good’ or ‘excellent’

- Q2: ‘all of the time’

- Q3: ‘all 7 days’

- Q4: ‘more than three months ago’, ‘never missed’ or ‘I’m not sure’

### Data and statistical analysis

Chi2 and Fisher-Exact tests were used to compare categorical data between groups and T-tests and Mann-Whitney tests for continuous data. For each adherence calculation we calculated specificity (i.e., percentage of patients with detectable VL which is identified as non-adherent), sensitivity (i.e., percentage of patients with undetectable VL which is identified as adherent), negative predictive value (NPV) (i.e., percentage of non-adherent patients that has a detectable VL), and positive predictive value (PPV) (i.e., percentage of adherent patients that has an undetectable VL). Specificity, sensitivity, NPV and PPV were determined for PRL and PR from ≤70% to ≤100%. With logistic regression analyses, independent risk factors for non-adherence calculated with PRL were determined. Risk factors for self-reported non-adherence within this patient population were previously reported [11]. Cut off values for pharmacy refill data adherence were set at 95% and 80%. The 95% cut off value is the gold-standard threshold for older cART regimens, the 80% cut off value is based on a study showing that this threshold appears sufficient to maintain virologic suppression with newer cART regimens [16]. Variables included in the analyses were: VL, gender, region of origin, sexual orientation, family situation, marital status, educational attainment, employment status, alcohol and substance use, social support, HIV-related stigma, treatment adherence self-efficacy, and physical and mental quality of life [11]. For these analyses, scores from the psychosocial variables (social support, internalized HIV-related stigma, treatment adherence self-efficacy, and physical and mental quality of life) were dichotomized based on their median values. In multivariable analysis, we submitted all variables demonstrating a *P*<0.15 in the univariable analyses.

## Results

### Patient population

In total, 301 MPLWH used cART for >6 months at inclusion. Of these patients two withdrew their informed consent for contacting their pharmacy, thus 299 patients were included. About 57% were male (n = 112), mean age was 43.2 (SD 10.2) and mean of years on cART was 4.5 (SD 3.3) (Table 1).The majority of the population originated from Sub Saharan Africa region (42.1%). Most of the patients were heterosexual (65.2%), lived with family (39.5%) and were unmarried (58.9%). Median CD4+ cell count was 550*10^6/L (IQR 420.0–710.0) and 11% had a detectable VL.

**Table 1:** Patient characteristics.

	Total	Complete pharmacy data	Incomplete pharmacy data	*P*
Variable	n = 299	n = 196	n = 103	
**Male (%)**	171 (57.2)	112 (57.1)	59 (57.3)	0.98^c^
**Age (mean, SD)**	43.2 (10.2)	44.9 (9.5)	39.9 (10.9)	<0.001^d^
**Years on cART (mean, SD)**	4.50 (3.3)	5 (3.3)	3.5 (3.1)	<0.001^d^
**cART regimen (%)**				<0.05^e^
*NRTI+NNRTI*	197 (65.9)	132 (67.3)	65 (63.1)	
*NRTI+PI*	87 (29.1)	50 (25.5)	37 (35.9)	
*NRTI+NNRTI+PI*	5 (1.7)	4 (2.0)	1 (1.0)	
*Other*^*a*^	10 (3.3)	10 (5.1)	0	
**Region of origin (%)**				0.84^c^
*Sub Saharan Africa*	126 (42.1)	83 (42.3)	43 (41.7)	
*The Caribbean*	62 (20.7)	38 (19.4)	24 (23.3)	
*Latin America*	59 (19.7)	39 (19.9)	20 (19.4)	
*Other*	52 (17.4)	36 (18.4)	16 (15.5)	
**Sexual orientation (%)**				0.74^c^
*Heterosexual*	195 (65.2)	130 (66.3)	65 (63.1)	
*Homosexual/Bisexual*	93 (31.1)	59 (30.1)	34 (33.0)	
*Doesn’t know*	8 (2.7)	6 (3.1)	2 (1.9)	
Missing data	3 (1.0)	1 (0.5)	2 (1.9)	
**Family situation (%)**				0.08^c^
*Lives alone*	110 (36.8)	76 (38.8)	34 (33.0)	
*Single parent*	49 (16.4)	31 (15.8)	18 (17.5)	
*Lives with family (parents*, *partner*, *kids)*	118 (39.5)	80 (40.8)	38 (36.9)	
*Other*	22 (7.4)	9 (4.6)	13 (12.6)	
**Marital status (%)**				0.57^c^
*Unmarried*	179 (58.9)	113 (57.7)	63 (61.2)	
*Married/registered partnership*	68 (22.7)	49 (25.0)	19 (18.4)	
*Divorced*	45 (15.1)	28 (14.3)	17 (16.5)	
*Widow/Widower*	9 (3.0)	5 (2.6)	4 (3.9)	
Missing data	1 (0.3)	1 (0.5)	0	
**Education (%)**				0.58^c^
*No formal/Primary school*	79 (26.4)	51 (26.0)	28 (27.2)	
*Secondary school*	94 (31.4)	61 (31.1)	33 (32.0)	
*Higher vocational school*	66 (22.1)	40 (20.4)	26 (25.2)	
*University*	58 (19.4)	42 (21.4)	16 (15.5)	
Missing data	2 (0.7)	2 (1.0)		
**Employment (%)**				0.19^c^
*Paid job/Self-employed*	119 (39.8)	83 (42.3)	36 (35.0)	
*Unemployed/Looking for a job*	82 (27.4)	50 (25.5)	32 (31.1)	
*Sick/Unable to work*	33 (11.0)	25 (12.8)	8 (7.8)	
*Other*	65 (21.7)	38 (19.4)	27 (26.2)	
**Alcohol and substance use (%)**				
*Alcohol use in past 30 days*	163 (54.5)	107 (54.6)	56 (54.4)	0.88^c^
Missing data	2 (1.9)			
*Substance use in past 30 days*	49 (16.4)	30 (15.3)	19 (18.4)	0.44^c^
Missing data	2 (1.9)			
**CD4-Count (10^6/L) (median, IQR)**	550.0 (420–710)	570 (440–720)	540 (350–680)	0.11^f^
**VL (%)**				
*HIV-1*	297 (99.3)	195 (99.5)	102 (99.0)	0.64^c^
*Detectable*^*b*^	33 (11.0)	19 (9.7)	14 (13.6)	0.31^c^
**Self-reported adherence (%)**				0.92^c^
*Adherent*	139 (46.5)	92 (46.9)	47 (45.6)	
*Non-adherent*	157 (52.5)	103 (52.6)	54 (52.4)	
Missing data	3 (1.0)	1 (0.5)	2 (1.9)	
**Psychosocial variables (median, IQR)**				
**Social support**	75.0 (42.2–90.6)	75.0 (40.6–87.5)	75.0 (43.8–93.8)	0.53^f^
Missing data	18 (6.0)	12 (6.1)	6 (5.8)	
**Stigma**	15.0 (12.0–18.0)	15.0 (12.0–18.8)	14.0 (12.0–18.0)	0.38^f^
Missing data	15 (5.0)	8 (4.1)	7 (6.8)	
**Self-efficacy**	8.8 (7.7–9.7)	8.9 (7.8–9.8)	8.8 (7.4–9.6)	0.31^f^
Missing data	50 (16.7)	3 (17.3)	16 (15.5)	
**Quality of life (QoL)**				
**Physical QoL**	52.3 (41.3–56.2)	52.5 (41.2–56.2)	51.3 (42.3–55.6)	0.89^f^
Missing data	8 (2.7)	4 (2.0)	4 (3.9)	
**Mental QoL**	49.4 (39.0–57.3)	49.0 (38.6–57.0)	49.9 (39.4–57.7)	0.80^f^
Missing data	8 (2.7)	4 (2.0)	4 (3.9)	

cART, combination Antiretroviral Therapy; NNRTI, non-nucleoside reverse transcriptase inhibitors; PI, protease inhibitors; INI, integrase inhibitors; NRTI/NtRTI, nucleoside reverse transcriptase inhibitors/ nucleotide reverse transcriptase inhibitors; VL, viral load; na, not applicable.

^a^ NRTI+INI (2), NNRTI+PI (2), NRTI only (2), PI only (4).

^b ^HIV-1 and HIV-2 > 50 copies/ml

^c ^Chi-square

^d ^T-test

^e ^Fisher`s Exact

^f ^Mann-Whitney test

Of the 299 patients, 245 provided one pharmacy address, 39 provided two pharmacy addresses, and four provided three pharmacy addresses. Patient pharmacies were unknown for 11 of the participants, because they could not remember the name of their pharmacy or were not known at the pharmacy that they had indicated. Pharmacies did not respond to repeated requests for data for 24 patients. For 31 patients data could not be used, for instance because medication was delivered at home. Of the remaining 233 patients, data about leftover medication was not available for 37, leaving for a total of 196 patients with complete data.

These 196 patients (complete data, CD) were significantly older (44.9 years, SD 9.5) than the 103 of whom the pharmacy refill data could not be fully retrieved (ID) (39.9 years, SD 10.9) (Table 1). In addition, the mean number of years on cART was significantly higher in the CD group (5 years) compared to the ID group (3.5 years). Regimens containing nucleoside reverse transcriptase inhibitors (NRTI’s) plus protease inhibitors (PI’s) were used less frequently in the CD group (25.5%) compared to the ID group (35.9%). Furthermore, patients in the CD group originated less often from the Caribbean (19.4% versus 23.3%). The two groups did not differ significantly in self-reported adherence percentages.

### Adherence using pharmacy refill data and self-reporting

Table 2 shows the number of patients who were classified as adherent with cut off values of ≤70% to ≤100%, for calculations including leftover medication (PRL), and for calculations excluding leftover medication (PR). Using a cut off value of ≤100% in the PRL group, 65.8% was classified as adherent compared to 44.6% in the PR group. Lowering the cut off value increased the percentage classified as adherent. Specificity was 52.6% for patients with PRL ≤100% and decreased with lower adherence cut off values. Higher cut off values had a lower sensitivity. PPV was around 93% for all cut off values, while NPV increased when lower cut off values were used.

**Table 2:** Comparison of adherence measures.

Adherence measure	Cutoff value	Adherent N (%)	Sensitivity (%)	Specificity(%)	PPV (%)	NPV (%)	OR (95% CI)
Pharmacy refill adherence when leftover medication in included **(PRL)** N = 196	≤ 100	129 (65.8)	67.8	52.6	93.0	14.9	2.34 (0.90–6.07)
	≤ 95	147 (75.0)	77.4	47.4	93.2	18.4	3.08 (1.17–8.11)
	≤ 90	154 (78.6)	80.8	42.1	92.9	19.0	3.06 (1.14–8.19)
	≤ 85	160 (81.6)	84.2	42.1	93.1	22.2	3.87 (1.43–10.48)
	≤ 80	168 (85.7)	88.7	42.1	93.5	28.6	5.71 (2.05–15.88)
	≤ 75	173 (88.3)	91.0	36.8	93.1	30.4	5.87 (2.03–17.01)
	≤ 70	176 (89.3)	92.1	31.6	92.6	30.0	5.37 (1.77–16.32)
Pharmacy refill adherence when leftover medication is excluded **(PR)** N = 233	≤ 100	104 (44.6)	45.3	61.9	92.3	10.0	1.35 (0.54–3.38)
	≤ 95	132 (56.7)	58.0	57.1	93.2	11.9	1.84 (0.74–4.56)
	≤ 90	157 (67.4)	68.9	47.6	92.9	13.1	2.01 (0.81–4.97)
	≤ 85	168 (72.1)	75.6	42.6	92.6	13.9	2.09 (0.84–5.23)
	≤ 80	183 (78.5)	80.2	38.1	92.9	16.0	2.49 (0.97–6.39)
	≤ 75	193 (82.8)	84.4	33.3	92.7	17.5	2.71 (1.02–7.23)
	≤ 70	198 (85.0)	92.4	28.6	93.7	17.1	2.52 (0.91–7.03)
**Self-reported adherence** N = 296		139 (47.0)	49.2	71.9	93.5	14.6	2.48 (1.11–5.56)
Self-reported adherence among patients with PRL N = 195^a^		92 (46.9)	49.2	72.2	94.6	12.6	2.51 (0.86–7.35)
Self-reported adherence among patients with PR N = 232^a^		109 (46.8)	48.6	70.0	94.5	11.4	2.21 (0.82–5.96)

Sensitivity, percentage of patients with undetectable viral load who are identified as adherent, Specificity, percentage of patients with detectable viral load who are identified as non-adherent; PPV, positive predictive value, percentage of adherent patients that has an undetectable viral load; NPV, negative predictive value, percentage of non-adherent patients that has a detectable viral load; PRL, pharmacy refill adherence when left over medication is included; PR, pharmacy refill adherence when left over medication is not included.

^a^ Self-reported adherence data from 1 participant was missing.

Using the self-reported adherence questions among the patients who had a self-reported adherence outcome (n = 296), 139/296 (47.0%) reported to be adherent (Table 2). When only participants with both a self-reported adherence outcome and a PRL outcome were included in the analyses, 92/195 (46.9%) reported to be adherent. Compared to the self-reported adherence measure, specificity was lower and sensitivity of the pharmacy refill measure was higher with decreasing PRL cut off values. There was no significant difference in PPV, and NPV between PRL using the higher cut off values and self-reported adherence.

### Factors associated with non-adherence

Factors associated with <95% pharmacy refill adherence in the univariable analyses were: having a detectable VL, having a ‘other’ type of employment status (vs paid employment), having a low physical quality of life, and having a low mental quality of live (data not shown). Variables demonstrating a *P*<0.15 were: experiencing low social support, and high internalized stigma. Factors associated with <80% pharmacy refill adherence were having a detectable VL, being on sick leave, having a ‘other’ type of employment status (vs paid employment), drug use in the previous 30 days, and having a low physical quality of life (data not shown). Being on a regimen containing a NRTI plus PI (vs a regimen containing NRTI plus NNRTI) demonstrated a *P*<0.15.

In the multivariable analyses (Table 3), having a ‘other’ type of employment status (vs paid employment), a low physical quality of life, and a low mental quality of life were marginally significantly associated with <95% pharmacy refill adherence (*P*<0.10). Having a detectable VL persisted to be significantly associated to a <80% pharmacy refill adherence, as was being on sick leave (vs. paid employment. Having an ‘other’ type of employment status (vs paid employment) was marginally significantly associated with <80% pharmacy refill adherence.

**Table 3:** Factors related to PRL non-adherence^a^.

Multivariable Regression	<95% PR adherence			<80% PR adherence		
Variable	OR	95% CI	*P*	OR	95% CI	*P*
**cART regime**						
*NRTI+NNRTI*				1		
*NRTI+PI*				1.16	0.42–3.21	0.78
*NRTI+NNRTI+PI*				0.75	0.06–10.37	0.83
*Other*				0	0	0.99
**HIV-RNA**						
*<50 copies/ml*	1			1		
*>50 copies/ml*	2.72	0.83–8.91	0.99	4.94	1.42–17.23	<0.05
**Employment status**						
*Paid employment*	1			1		
*Unemployed*	1.34	0.45–3.95	0.60	2.09	0.58–7.60	0.26
*On sick leave*	1.92	0.54–6.78	0.31	4.76	1.10–20.66	<0.05
*Other*	3.53	1.23–10.11	<0.05	3.41	0.89–13.06	0.07
**Drugs use < 30 days**						
*No*				1		
*Yes*				2.31	0.79–6.76	0.13
**Social support**						
*High social support*	1					
*Low social support*	1.28	0.56–2.92	0.56			
**Internalized HIV-related stigma**						
*Low internalized stigma*	1					
*High internalized stigma*	1.56	0.71–3.43	0.27			
**Quality of life**						
*High physical QoL*	1			1		
*Low physical QoL*	2.26	0.97–5.23	0.06	1.54	0.57–4.14	0.40
*High mental QoL*	1					
*Low mental QoL*	1.82	0.93–3.57	0.08			

Educ, Education; Prim, Primary; QoL, Quality of Life.

^a^All variables with a *P*<0.15 in the univariable analyses were submitted in multivariable analyses.

## Discussion

The HIV-infected population in many western European countries increasingly consists of migrants from countries where HIV-infection is highly prevalent. Practical methods to assess adherence in this population are needed as migrants are at risk of having a lower adherence and suboptimal virological response. Our study demonstrates that pharmacy refill adherence and four self-report adherence questions, can predict undetectable VL. Self-reported adherence questions have higher specificity and lower sensitivity compared to PRL. PPV and NPV are similar for both methods. In addition, having an ‘other’ type of employment status is associated with <95% and having a detectable VL with 80% pharmacy refill adherence.

HIV-RNA was detectable in 11% of HIV-infected migrants on cART. This percentage of viremic patients is similar to that found in other studies measuring the association between adherence and virological failure [21, 22], but is lower than previously found in Dutch MPLWH [6]. It is not clearly defined what the gold-standard for adherence studies in HIV-infected patients is. Some studies use pharmacy refill data as golden standard to asses self-reported adherence [23], but most studies use virologic failure [6, 16, 21, 24]. However, incomplete adherence is associated with residual HIV-1 replication, even in the absence of plasma viremia [25]. Because the clinical goal of treatment with cART is to achieve or maintain undetectable plasma viral load, we compared self-reported- and pharmacy refill adherence in their ability to predict viral suppression.

An association between having an ‘other’ type of employment status with <95% pharmacy refill adherence was found. A total of 38 patients were in this group, of whom 15 were identified to be <95% adherent. Most reported to be a housewife/-man (n = 7) or student (n = 4). Being on sick leave was associated with <80% pharmacy refill adherence. A total of 25 were in this group, of whom 6 were identified to be <80% adherent. A possible reason for these associations might be a less structured lifestyle, although this remains speculative.

Of the patients showing 100% PRL adherence, 21.2% was misclassified as non-adherent when leftover medication was not taken into account. This supports the results from another study where a high percentage was misclassified as non-adherent [13]. Therefore we advise to include leftover medication from previous refills in future studies calculating adherence to cART based on pharmacy refill data in countries that do not have a closed pharmacy system.

Defining adherence using pharmacy refill data is challenging. With older cART regimens, a threshold of 95% has been used in clinical practice. Recently it has been shown that for three regimens (emtricitabine-tenofovir plus efavirenz or raltegravir or boosted protease inhibitor (darunavir or atazanavir)) 80% adherence resulted in no more than 3.5% virological failure, whereas adherence of >90% resulted in 1.1% virological failure [16]. The majority of our patients (n = 284, 95%) used a nucleoside reverse transcriptase inhibitor (NRTI) plus a non-nucleoside reverse transcriptase inhibitor (NNRTI) or protease inhibitor (PI) (Table 1) and our results support the suggestion that a pharmacy refill adherence of 80–90% might be sufficient to maintain virologic suppression with the newer cART regimens.

Self-reported adherence is a cheap and easy way to measure adherence. Self-reported adherence using elaborate questionnaires with over 19 items has been shown to predict virological suppression in HIV-patients in high resource settings [6, 16]. In low resource settings, self-reported adherence questions similar to those used in the present study were reported to predict detectable viral load [14]. However, a questionnaire with a visual analog score scale for missed doses and 2 questions regarding missed doses [26] was found to be inferior to pharmacy refill data in predicting virologic failure [22]. Little is known about the validity of self-reported adherence among migrant populations. Self-reported adherence may be biased due to cultural differences or language barriers. Using different questionnaires for adherence, migrants using cART showed a tendency to provide more socially desirable answers [6]. Social desirability bias could even be more pronounced if adherence questions are administered during face-to-face interviews due to literacy problems. However, using a 4-item questionnaire, we show that self-reported adherence predicts virologic suppression in MPLWH treated in a high resource setting. In MPLWH the 4-item questionnaire seems an easy way to identify non-adherent patients and to further determine adherence and barriers to adherence.

The strength of our study is that it is a real life study involving MPLWH using cART during a wide range of time. Our results may be extrapolated to similar clinical settings. Our study should be viewed in the context of some limitations. We measured adherence, but not the barriers to adherence. In an analysis of 11 AIDS Clinical Trials Group studies it was shown that patients reporting a higher number of adherence barriers had lower odds of attaining virologic suppression [27]. The most commonly mentioned adherence barriers were being away from home or forgetting to take the pills. Adherence barriers may differ between different HIV-infected populations. In MPLWH, internalized stigma was associated with non-adherence [10]. Therefore, after identification of MPLWH that are non-adherent, barriers should be assessed to start successful interventions. Another limitation is that we did not evaluate whether non-adherence is associated with the use of certain cART regimens. However, most patients in the Netherlands, including migrants, use the newer cART regimens and since we wanted to study our adherence measures in real life, we choose not to break the results down to the different cART regimens.

In conclusion, in MPLWH both methods, PRL and self-reported adherence questions, can predict undetectable VL. Using self-reported adherence questions results in a lower sensitivity and a higher specificity compared to PRL. PPV and NPV are similar for both methods. Collecting PRL of MPLWH in a country with no closed pharmacy system is laborious and not possible for all patients. This study shows that using four items in self-reported adherence is sufficient to predict adherence which is crucial for optimal clinical outcome in HIV-infected migrants.
